# Continuing Professional Development – Medical imaging

**DOI:** 10.1002/jmrs.621

**Published:** 2022-11-01

Maximise your CPD by reading the following selected article and answer the five questions. Please remember to self‐claim your CPD and retain your supporting evidence. Answers will be available via the QR code and online at www.asmirt.org/news-and-publications/jmrs, as well as published in JMRS – Volume 70, Issue 4 December 2023.

## Medical Imaging – Original Article

### IMPACT (Information Medically Pertinent in Acute Computed Tomography) requests: Delphi study to develop criteria standards for adequate clinical information in computed tomography requests in the Australian emergency department

Castillo C, Steffens T, Livesay G, Sim L, Caffery L. (2022) *J Med Radiat Sci*. https://doi.org/10.1002/jmrs.607

1. What do the authors of this study see as the next step in improving clinical information in CT requests?
  - Education of referrers on essential information for referring clinicians
  - Develop artificial intelligence tools to interrogate the electronic health record (EHR) for the required information
  - Encourage radiographers to conduct a full assessment of the patient prior to scanning
  - Encourage radiologists to review the electronic health record (EHR) for every patient
2. Which of the following is most correct? Delphi studies are used to
  - Generate discussion among a group of people
  - Gain a consensus among a panel of experts
  - Facilitate online focus groups
  - Observe participants and collect data using researchers' five senses
3. Which of the following was an essential item of clinical information for CT Chest scan referrals?
  - Smoking history
  - Presence of fever/febrile
  - Duration of presenting symptoms
  - Human immunodeficiency virus (HIV)/blood‐borne virus (BBV) status
4. Which of the following was an essential item of clinical information for CT Abdomen referrals?
  - Medication history
  - Recent travel history
  - Family history of cancer
  - History of prior abdominal pathology
5. Which of the following was an essential item of clinical information for CT Multi‐Trauma (CT Trauma Series) referrals?
  - Full blood count
  - Localising neurology
  - History of intravenous drug use
  - History of emergency department presentations

**Recommended further reading:**

1. Boulkedid R, Abdoul H, Loustau M, Sibony O, Alberti C. Using and Reporting the Delphi Method for Selecting Healthcare Quality Indicators: A Systematic Review. Wright JM, editor. PLoS ONE [Internet]. 2011 Jun 9;6(6): e20476. Available from: https://www.ncbi.nlm.nih.gov/pmc/articles/PMC3111406/.
2. Castillo C, Steffens T, Sim L, Caffery L. The effect of clinical information on radiology reporting: A systematic review. Journal of Medical Radiation Sciences. 2020 Sep;68(1). Available from: https://onlinelibrary.wiley.com/10.1002/jmrs.424.
3. Bardach SH, Real K, Bardach DR. Perspectives of healthcare practitioners: An exploration of interprofessional communication using electronic medical records. Journal of Interprofessional Care. 2017 Feb 2;31(3):300–6. Available from: https://www.ncbi.nlm.nih.gov/pmc/articles/PMC5896008/.

## Answers


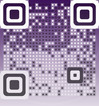


Scan this QR code to find the answers, or visit www.asmirt.org/news-and-publications/jmrs

